# Resveratrol Ameliorates Trigeminal Neuralgia-Induced Cognitive Deficits by Regulating Neural Ultrastructural Remodelling and the CREB/BDNF Pathway in Rats

**DOI:** 10.1155/2022/4926678

**Published:** 2022-11-28

**Authors:** Li Zhang, Bijia Song, Xuan Zhang, Mu Jin, Lixin An, Tiandong Han, Fan Liu, Zhiyao Wang

**Affiliations:** ^1^Department of Anesthesiology, Beijing Friendship Hospital, Capital Medical University, Beijing 100012, China; ^2^Department of Anesthesiology, Beijing Jingmei Group General Hospital, Beijing 102300, China; ^3^Department of Urology, Beijing Friendship Hospital, Capital Medical University, Beijing 100012, China; ^4^National Human Brain Bank for Development and Function, Institute of Basic Medical Sciences, Chinese Academy of Medical Sciences, School of Basic Medicine, Peking Union Medical College, Beijing 100005, China; ^5^Department of Anesthesiology, Zhongshan Hospital, Fudan University, Shanghai 200032, China

## Abstract

Chronic pain often leads to cognitive impairment. Resveratrol (Res), a natural polyphenol existing in Polygonum cuspidatum, has been widely investigated for its antinociceptive, anti-inflammatory, and neuroprotective properties. Our aim was to explore the ameliorating effects of resveratrol on pain-related behaviors and learning and memory deficits induced by cobra venom-induced trigeminal neuralgia (TN). The TN model of rats was established by injecting cobra venom solution beneath the epineurium of the infraorbital nerve. Resveratrol was intragastrically administered at a dose of 40 mg/kg twice daily beginning on postoperative day 15. CREB inhibitor 666-15 was intraperitoneally administered at a dose of 10 mg/kg from POD 35-42 after morning resveratrol treatment. Mechanical allodynia was measured via von Frey filaments. Rat free movement was videotaped and analyzed. Spatial learning and memory were evaluated via the Morris water maze test. Ultrastructures of the hippocampal DG region and infraorbital nerve were observed by transmission electron microscopy. We found that resveratrol alleviated TN-induced allodynia, ameliorated learning and memory deficits, restored the ultrastructure of hippocampal neurons and synapses, repaired the damaged myelin sheath of the infraorbital nerve, and activated the CREB/BDNF pathway in the hippocampus of TN rats. CREB inhibitor administration suppressed the resveratrol-rescued abnormal hippocampal ultrastructural changes and aggravated spatial learning and memory impairment by inhibiting CREB/BDNF pathway activation in the hippocampus. Our findings indicated that resveratrol alleviated pain and improved cognitive deficits, probably by regulating neural ultrastructure remodelling and the CREB/BDNF pathway.

## 1. Introduction

Chronic pain is one of the major symptoms of various neurodegenerative diseases and usually leads to cognitive impairment. Processed by a complex neuronal network, chronic pain can cause alterations in neuroanatomy as well as cognitive capacities by changing the function and structure of the central nervous system [1–3]. Clinical patients with chronic pain are often comorbid with emotional and cognitive disorders [4], and patients with cognitive diseases often suffer from allodynia and hyperalgesia [5], which exacerbates the vicious cycle between pain and cognitive disorders. Therefore, developing a novel and effective therapy for cognitive impairments associated with chronic pain remains an urgent issue.

Previous research has shown that chronic pain consistently activates brain regions involved in emotional and cognitive pain processing [6]. Given that the hippocampus is a critical brain region responsible for encoding and consolidating memory, experiencing pain, and other cognitive functions, hippocampal plasticity in chronic pain has been intensively studied [7]. Accumulating evidence has demonstrated that hippocampal structure and function exhibit impairments in various cognitive deficits [8]. Our previous study also indicated that hippocampal neurons and synaptic morphology were altered in a trigeminal neuralgia rat model, which also showed cognitive deficits [9]. Research from other groups found that chronic neuropathic pain caused memory deficits by altering hippocampal neuronal structure and synaptic plasticity [10]. Although the hippocampus has been significantly involved in pain and cognitive deficits, the molecular mechanisms by which nociceptive signals are transmitted to hippocampal regions and contribute to synaptic plasticity alterations remain unclear.

cAMP-response element-binding protein (CREB) is a key transcription factor that mediates synaptic nociceptive signaling transmission and regulates gene transcription [11]. CREB is activated by phosphorylation at S133, which migrates into the nucleus and causes the transcription of target genes, such as brain-derived neurotrophic factor (BDNF). BDNF plays a key role in the formation and development of pain, cognition, and memory [12–14]. The CREB/BDNF pathway is critical for both pain and cognition. Our previous study also demonstrated that the hippocampal CREB/BDNF pathway was downregulated in TN-induced cognitive deficits [9]. However, little is known about how the CREB/BDNF pathway in the hippocampus is involved in neuropathic pain and related cognitive deficits.

Due to a lack of knowledge about the mechanisms of chronic pain-induced cognitive deficits, treatments for improving this kind of cognitive deficit are very limited. Resveratrol, a polyphenol, is a major active component of Polygonum cuspidatum found in a wide variety of plants, such as red grapes and blueberries [15]. Multiple biological benefits have been found from resveratrol, such as antioxidation, anti-inflammation, analgesia, and neuroprotection [16–18]. Neuroprotection from resveratrol can improve cognitive impairments in different experimental models [19, 20]. Meanwhile, resveratrol can relieve pain caused by trigeminal neuralgia (TN) and other neuropathies [21–23]. Its neuroprotective and analgesic effects suggest that resveratrol may be effective for treating cognitive deficits induced by chronic pain. However, the potential mechanisms remain to be clarified.

In the present study, chronic resveratrol treatment significantly alleviated pain and ameliorated TN-induced cognitive deficits by restoring the ultrastructure of hippocampal neurons and synapses and upregulating the CREB/BDNF pathway in the hippocampus.

## 2. Materials and Methods

### 2.1. Animals

All animal procedures performed in this study were approved by the Animal Ethical Committee of Beijing Friendship Hospital, Capital Medical University (#15-1002 and #22-1001), according to the guidelines of the International Association for the Study of Pain (IASP). Adult male Sprague–Dawley rats (180-200 g) were used in this study and were housed (4 rats per cage) in a standard 12-hour light/dark cycle with food and water available ad libitum. Rats were provided with standard chow (REG; NIH-31; Harlan Teklad, Madison, WI, USA) and sterile water [24]. Experiments were performed between 9 : 00 am and 16.00 pm in temperature- and humidity-regulated rooms (22-24°C, relative humidity: 60-70%).

### 2.2. Animal Model of Trigeminal Neuralgia

A rat trigeminal neuralgia model was established as described in previous studies [25, 26]. In brief, rats were anesthetized by sodium pentobarbital (40 mg/kg, i.p.). After exposing the left infraorbital nerve, 4 *μ*L of cobra venom (Formosan cobra; Sigma–Aldrich Co., USA) solution (100 mg/ml in saline) was injected into the subepineurium of the infraorbital nerve. The animals in the sham surgery group were injected with saline. Rats (*n* = 16 per group) were randomly assigned to the sham group (normal saline nerve injection), TN group (trigeminal neuralgia induced by intranerve injection of cobra venom), TN + Veh group (trigeminal neuralgia induced by intranerve injection of cobra venom + vehicle (2% DMSO saline)), TN + Res group (trigeminal neuralgia + resveratrol treatment), TN + Res + CREB group (trigeminal neuralgia + resveratrol treatment + CREB inhibitor), and TN + Res + Veh group (trigeminal neuralgia + resveratrol treatment + vehicle (2% DMSO saline)).

### 2.3. Drug Preparation

Resveratrol (Sigma–Aldrich, USA) was dissolved in saline mixed with 5% DMSO and given in a volume of 0.2 ml per 100 g body weight of the rats. Resveratrol (40 mg/kg) was administered by gavage twice a day (morning and evening) from postoperative day (POD) 15 to POD 42 based on previous literature [27–30]. The vehicle group received 2% DMSO saline treatment only. CREB inhibitor 666-15 (Cat No. HY-101120, MCE, USA) was administered by intraperitoneal injection from POD 35 to POD 42 after morning resveratrol treatment according to the dose of 10 mg/kg in the literature [31, 32].

### 2.4. Behavioral Assessment of Pain

Mechanical allodynia was assessed by the updown method using von Frey filaments (Stoelting) [33]. Rats (*n* = 16 per group) were allowed to acclimate in suspended cages with a wire mesh floor. The filaments were applied to the ipsilateral infraorbital nerve territory near the center of the vibrissal pad and held for approximately 6~8 s. Brisk face withdrawal or head turning was considered a positive response. Spontaneous behaviors such as face grooming and exploration (walking, running, climbing, or rearing) were recorded to evaluate spontaneous changes in rat activity as previously described [9]. Both spontaneous behavior recordings and pain behavioral tests were performed in the morning 1 h before the administration of resveratrol.

### 2.5. Morris Water Maze (MWM) Test

As previously described [34], spatial learning and memory abilities were evaluated by the MWM test 4 weeks after resveratrol administration (6 weeks after surgery, from POD 43 to 47 after surgery). The MWM test comprised 2 phases: an acquisition trial and a probe trial. The acquisition trial was performed from POD 43 to 46 after surgery, and the probe trial was performed on POD 47 after surgery. The MWM test was performed in a black circular tank (180 cm diameter and 60 cm depth) filled with opaque water (19-22°C) containing an escape platform (10 cm diameter and 2 cm beneath the water) [35–38]. The tank was suffused with a 30 cm-deep water level, which was mixed with 30 ml of ink to obscure visual cues [35]. The platform was painted black as the pool wall and floor to make it invisible in the water. The water tank was divided into 4 quadrants and surrounded by 4 visible external cues in the wall. The rat was placed into the water facing the tank at different predetermined positions in each trial and allowed to swim no longer than 120 s to arrive at the escape platform, which always remained in the same position. Rats that failed to arrive at the escape platform within 120 s were placed on the platform and allowed to rest there for 20 s. Escape latency and swimming speed were recorded by videotaping. Each rat was tested in 4 acquisition trials with 20-minute intervals for 4 consecutive days. On day 5, a probe trial was performed to evaluate the spatial retention ability. The rat was put into the tank from the north to the place of the escape platform, which had been removed, and allowed to swim for 120 s. The percentage of time that the rats spent in and the number they crossed over the target quadrant (the quadrant that previously contained the escape platform) were counted. All behavioral tests and measurements were performed by the researchers who were blinded to the group assignment. The performance of the rat was tracked automatically using a video tracking system (SMART 3.0 system: Panlab, Harvard Apparatus).

### 2.6. Electron Microscopy

On POD 48, rats (*n* = 8 per group) were anesthetized with pentobarbital sodium (40 mg/kg, i.p.) and perfused via the left ventricle with saline followed by 2% glutaraldehyde and 4% paraformaldehyde in PBS (Sigma–Aldrich). The hippocampus and the left infraorbital nerve were collected and postfixed in 3% glutaraldehyde for 2 h. After washing in 0.1 M phosphate buffer (PB) three times, the specimens were postfixed in 1% osmium tetroxide for 2 h, dehydrated in graded ethanol, embedded in araldite, and cut into 50–70 nm ultrathin sections for transmission electron microscopy observation (TEM; H-9000NARI baraki; Hitachi, Japan).

### 2.7. Western Blots

Western blotting was performed as described previously [39–41]. The hippocampus was collected from deeply anesthetized rats (*n* = 8 per group, 40 mg/kg, i.p.) and snap-frozen in liquid nitrogen. Tissues were homogenized on ice in RIPA buffer containing protease inhibitor cocktail and phosphatase inhibitor cocktail (Sigma–Aldrich). After centrifugation, the supernatants were collected and denatured with SDS–PAGE loading buffer for 5 min at 95°C. Equal amounts of protein were separated by SDS–PAGE and transferred to PVDF membranes (GE Healthcare Life Science). After blocking with 5% nonfat milk for 1 h at room temperature, membranes were incubated with primary antibodies overnight at 4°C (rabbit anti-pCREB, 1 : 1000, rabbit anti-CREB, 1 : 1000, Cell Signaling Technology; rabbit anti-BDNF, 1 : 600, Abcam). Then, the membranes were washed with TBST and incubated with secondary antibodies (1 : 1000, Santa Cruz Biotechnology) for 1 h at room temperature. Finally, bands were detected with an enhanced chemiluminescence reagent eECL Kit (CWBio, China) and quantified using ImageJ software (NIH, USA). GAPDH (rabbit antibody, 1 : 5000, Abcam) was used as the loading control.

### 2.8. Immunofluorescence Staining

As previously described [40, 41], rats (*n* = 8 per group) were anesthetized with sodium pentobarbital (40 mg/kg, i.p.) and perfused with PBS followed by fresh 2% glutaraldehyde and 4% paraformaldehyde through the ascending aorta. The brain was collected and fixed in 4% paraformaldehyde for 4 h and then dehydrated in 30% sucrose overnight at 4°C. The brain tissues were embedded in the optimal cutting temperature compound and finally cut to a thickness of 15 *μ*m in a -20°C cryostat for immunofluorescent staining. After blocking in 10% normal goat serum and 0.2% Triton X-100 in PBS for 1 h at room temperature, the tissue sections were incubated overnight at 4°C in 10% normal goat serum in PBS containing primary antibodies such as rabbit anti-pCREB, 1 : 150, Cell Signaling Technology; rabbit anti-BDNF, 1 : 100, Abcam; mouse anti-Neun, 1 : 300, Abcam; mouse anti-GFAP, 1 : 500, Abcam; and mouse anti-Iba1, 1 : 500, Abcam. Afterwards, these slices were incubated with Alexa Fluor 594-conjugated goat anti-rabbit secondary antibodies and Alexa Fluor 488-conjugated goat anti-mouse secondary antibodies (1 : 600, Jackson ImmunoResearch) for 1 h. Then, they were washed in PBS and cover-slipped with VECTASHIELD Mounting Medium with DAPI (Cat: H-1200, Vector Lab). Images were captured by a microscopic imaging system (Olympus BX61 and FluoView).

### 2.9. Statistical Analysis

Data are expressed as the mean ± standard error of the mean (mean ± SEM) and were analyzed using GraphPad 5 and SPSS software 17.0. For analysis of behavioral alterations, latencies to reach the platform, and swimming speeds in the MWM, data were analyzed with repeated measures 2-way ANOVA with Bonferroni's post-hoc test. Data on the percentage of time spent in the platform quadrant and platform crossings were analyzed with one-way ANOVA. Data on western blot, immunohistochemistry, and the ultrastructural changes of the hippocampus were analyzed with one-way ANOVA followed by Student-Newman–Keuls test. *P* < 0.05 was considered statistically significant.

## 3. Results

### 3.1. Resveratrol Reversed Cobra Venom-Induced Abnormal Spontaneous Behaviors and Mechanical Allodynia in Rats

**Figure 1:**
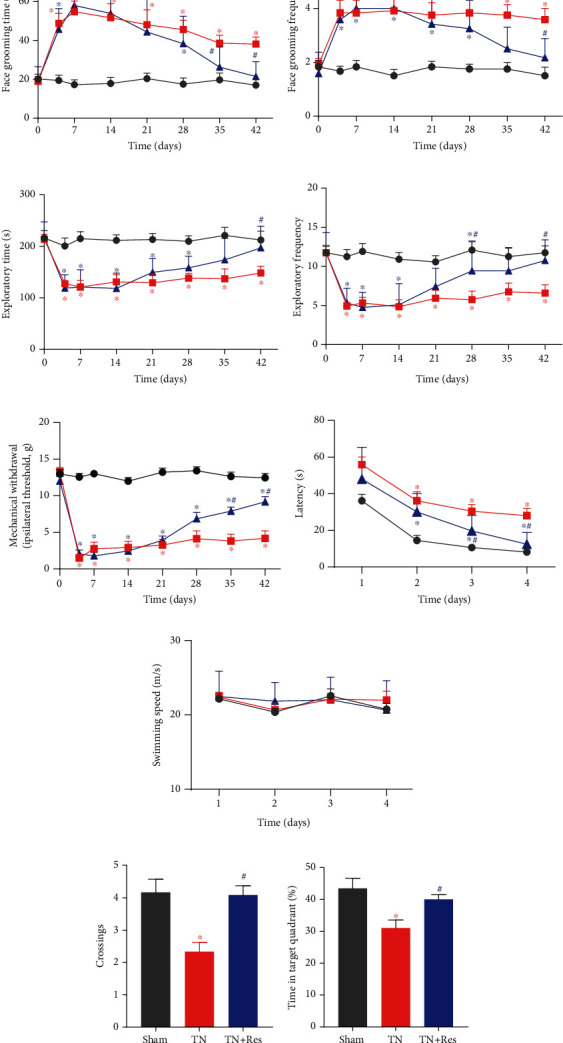
Behavioral alterations in different experimental rats after resveratrol treatment. (a, b) Duration (a) and frequency (b) of face-grooming behaviors at different time points. (c, d) Duration (c) and frequency (d) of exploratory activities at different time points. (e) Ipsilateral mechanical allodynia at different time points. (f) The average escape latency in the first four days of the Morris water maze (acquisition trials). (g) Swimming speeds in the first four days of the Morris water maze (acquisition trials). (h) The number of platform site crossings in different groups during probe trials. (i) The times in the target quadrant in different groups during probe trials. Data are shown as the mean ± SEM (*n* = 16 per group). Repeated measures 2-way ANOVA with Bonferroni's post-hoc test was used to analyze behavioral alterations, latencies to reach the platform, and swimming speeds in the MWM. Data on the percentage of time spent in the platform quadrant and platform crossings were analyzed with one-way ANOVA. ^∗^*P* < 0.05 vs. sham. ^#^*P* < 0.05 vs. TN + Veh.

Starting on POD 15, rats in the TN group were intragastrically administered with resveratrol (40 mg/kg, in 2% DMSO saline, TN + Res) or vehicle (2% DMSO saline, TN + Veh) twice a day for 4 weeks. As a result of myelin sheath damage in the infraorbital nerve, face-grooming activities were significantly increased in both duration (Figure 1(a)) and frequency (Figure 1(b)) in the rats receiving intraorbital nerve injection of cobra venom (TN + Veh group). Meanwhile, the duration (Figure 1(c)) and frequency (Figure 1(d)) of their exploratory behaviors were markedly decreased compared to those of the sham group. The abnormally increased face-grooming and decreased exploratory activities were significantly rectified by resveratrol treatment compared to the vehicle (2% DMSO saline)-treated group on POD 42 (Figures 1(a)–1(d)). In addition to abnormal spontaneous behaviors, rats in the TN + Veh group also displayed significant facial mechanical allodynia after cobra venom injection, which lasted for 42 days and was significantly attenuated by resveratrol 4 weeks of treatment (Figure 1(e)). Mechanical sensitivity was not changed in the facial area of rats in the sham group.

### 3.2. Resveratrol Improved Cobra Venom-Induced Spatial Learning and Memory Impairment in Rats

To investigate whether chronic pain changes cognitive functions in TN rats, we used the MWM test to evaluate their spatial learning and memory abilities. As shown in Figure 1(f), during 4-day acquisition trials (POD 43-46, after spontaneous behaviors and mechanical allodynia tests), rats in the TN + Veh group took significantly more time to find the escape platform in the 4 days (POD 43-46) of acquisition trials than rats in the sham surgery group. On the other hand, rats in the TN + Res group took significantly less time to arrive at the escape platform in the trials on day 4 than rats in the TN + Veh group (Figure 1(f)). There was no significant difference in swimming speed among the 3 groups (Figure 1(g)). Rat memory retention capacity was examined in the probe trials on day 5, when the escape platform was removed. The number of rats crossing the site where the platform was placed and the percentage of time that they spent in the target quadrant were recorded and compared. Rats in the TN + Veh group displayed fewer chances to cross the platform site and spent less time in the target quadrant while swimming in the tank than rats in the sham group, suggesting that rats in the TN + Veh group were suffering learning and memory impairments (Figures 1(h) and 1(i)). On the other hand, resveratrol significantly improved the impaired memory retention ability in rats in the TN + Res group and increased the time they spent in the target quadrant.

### 3.3. Resveratrol Rescued Cobra Venom-Induced Abnormal Ultrastructural Changes in the CA1 Region of the Hippocampus

**Figure 2:**
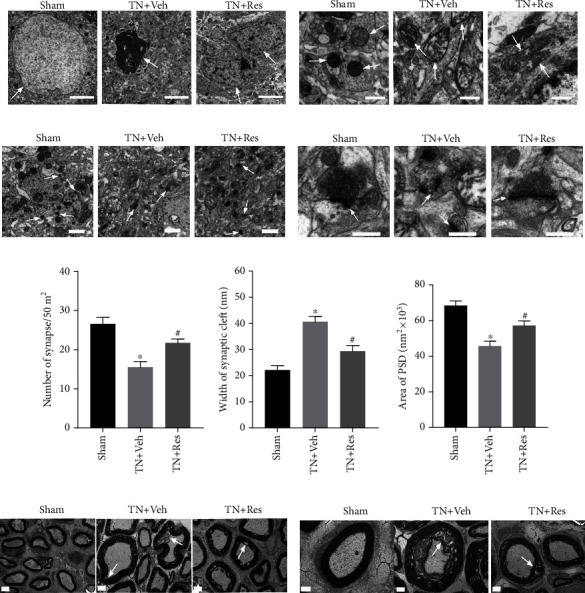
Abnormal ultrastructural changes in neurons and synapses in the DG region of the hippocampus, as well as infraorbital nerves, after resveratrol treatment. (a) Images illustrating the differences in nuclei (white arrow) in the sham, TN + Veh, and TN + Res groups (scale bars = 5 *μ*m). (b) Images illustrating the differences in mitochondria (white arrow) in the sham, TN + Veh, and TN + Res groups (scale bars = 0.5 *μ*m). (c) Images illustrating the differences in synapse density in the sham, TN + Veh, and TN + Res groups (magnification ×3000, scale bars = 1 *μ*m). White arrows indicate the synapses. (d) Images illustrating the differences in synapse cleft, presynaptic vesicles, and PSD in the sham, TN + Veh, and TN + Res groups (magnification ×10000, scale bars = 0.5 *μ*m). White arrows show the synaptic linkages. (e) Number of synapses in the DG region of the hippocampus. (f) Width of the synaptic cleft in the hippocampal DG region. (g) Area of postsynaptic density (PSD) in the hippocampal DG region. (h) Low magnification images showing the ultrastructural changes in the infraorbital nerves (ION) of rats in the sham, TN + Veh, and TN + Res groups (magnification ×1500, scale bars = 2 *μ*m). White arrows indicate demyelination and loss of axons, accompanied by disturbed myelin structure and altered layers. (i) High magnification images illustrating the ultrastructural changes in the IONs of rats in the sham, TN + Veh, and TN + Res groups (magnification ×3000, scale bars = 1 *μ*m). Data are expressed as the mean ± SEM (*n* = 8 per group). Data analysis was performed using one-way ANOVA. ^∗^*P* < 0.05 vs. sham. ^**#**^*P* < 0.05 vs. TN + Veh.

The hippocampus is critical for normal cognitive function. To investigate whether the impaired learning and memory ability observed in rats in the TN + Veh group was correlated with any structural changes in the hippocampus, we next checked the ultrastructure in the hippocampus using TEM (Figure 2). In the sham group, hippocampal neuron nuclei were large and round, containing a uniform density of chromatin. Their mitochondria had smooth membranes and regularly arranged mitochondrial cristae in the cytoplasm. However, in TN + Veh rats, the hippocampal neurons displayed irregular clumping of chromatin in the nucleus, swollen mitochondria with irregular shapes, and decreased numbers of cristae. After treatment with resveratrol, the ultrastructure of hippocampal neurons in TN + Veh rats was normalized, as indicated by more organized nuclei and mitochondria (Figures 2(a) and 2(b)). The morphology and number of synapses from hippocampal neurons were also changed in TN + Veh rats compared with sham surgery rats (Figures 2(c) and 2(d)). The normal morphology of synapses in the hippocampus from rats in the sham group was evidenced by a clear and intact membrane outline, abundant presynaptic neurotransmitter vesicles, and thick postsynaptic density (PSD) under the postsynaptic membrane. On the other hand, the synapses in TN + Veh rats no longer maintained a clear membrane outline. Presynaptic neurotransmitter vesicles were decreased. The PSD was thinner than normal under the postsynaptic membrane. The damaged synapses in the hippocampus of the rats in the TN + Veh group were rescued by resveratrol treatment, as indicated by increased presynaptic vesicle density and thickened PSD (Figures 2(c) and 2(d)). Compared with rats in the sham group, the number of synapses in TN + Veh rats decreased significantly, accompanied by an increased width of the synaptic cleft and a reduced area of PSD. Resveratrol treatment rescued the aberrant morphology and number of synapse changes in hippocampal neurons. The number of synapses in TN + Res rats increased remarkably, along with a shortened width of the synaptic cleft and an increased area of PSD compared with TN + Veh rats (Figures 2(e) and 2(g)).

### 3.4. Resveratrol Repaired Cobra Venom-Induced Abnormal Ultrastructural Changes in the Infraorbital Nerve

To confirm whether cobra venom induced demyelination in large and medium fibers in the infraorbital nerve, the ultrastructure of the infraorbital nerve was examined by TEM and compared between different groups of rats (Figures 2(h) and 2(i)). Normally, myelinated nerve fibers were observed in the cross-section of infraorbital nerves from the rats in the sham group. Severe demyelination and loss of axons were found in the infraorbital nerves of the rats in the TN + Veh group, as demonstrated by destroyed myelin sheaths and expanded concentric myelin layers, suggesting that injecting cobra venom into the infraorbital nerve induced trigeminal neuralgia. We also found that the damaged myelin sheath was repaired after treatment with resveratrol. Compared with TN + Veh rats, the demyelination changes in the infraorbital nerves of the rats in the TN + Res group were alleviated, as demonstrated by rescued myelin sheaths and less expanded concentric myelin layers, indicating that resveratrol treatment could attenuate abnormal ultrastructural changes in the infraorbital nerves induced by cobra venom (Figures 2(h) and 2(i)).

### 3.5. Resveratrol Activated the CREB/BNDF Pathway in the Hippocampus

**Figure 3:**
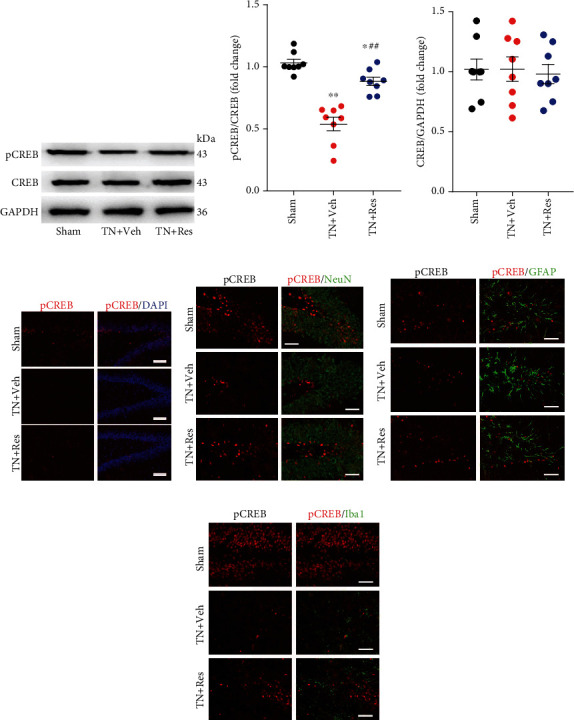
Expression and cellular distributions of pCREB protein in the DG region of the rat hippocampus. (a) Western blot showing the expression of pCREB protein in different experimental rats. (b) Quantitative analysis showing the ratio of pCREB/CREB in the hippocampal DG region from different experimental rats. (c) Quantitative analysis showing the ratio of CREB/GAPDH in the hippocampal DG region from different experimental rats. (d) Immunostaining showing the coexpression of pCREB with DAPI in the hippocampal DG region from different experimental rats. (e) Immunostaining showing the coexpression of pCREB with Neun in the hippocampal DG region from different experimental rats. (f) Immunostaining showing the coexpression of pCREB with GFAP in the hippocampal DG region from different experimental rats. (g) Immunostaining showing the coexpression of pCREB with Iba1 in the hippocampal DG region from different experimental rats. In (b, c), each column represents the mean ± SEM. (*n* = 8 rats per group). Data analysis was performed using one-way ANOVA. ^∗^*P* < 0.05, ^∗∗^*P* < 0.01 vs. sham. ^##^*P* < 0.01 vs. TN + Veh.

The CREB/BDNF pathway has been suggested to be involved in synaptic plasticity and spatial learning and memory [9]. We next analyzed the CREB/BDNF pathway change in the hippocampus after cobra venom intranerve injection. Our western blot results showed that TN significantly decreased the phosphorylation of CREB (pCREB) in the hippocampus compared to that in the sham group. Resveratrol treatment (40 mg/kg twice daily for 4 weeks) reversed the TN-induced decrease in the level of pCREB in the hippocampus of rats (Figures 3(a) and 3(b)). The level of CREB proteins was not significantly changed in the hippocampus after cobra venom intranerve injection and resveratrol treatment (Figures 3(a) and 3(c)). Similar to the western blot results, immunofluorescence staining (IF) revealed that the decreased level of pCREB was reversed by resveratrol treatment in the hippocampal tissue of TN rats (Figure 3(d)). Then, we characterized the expression profile of pCREB in the hippocampus. Double IF results showed that the pCREB protein mainly colocalized with Neun (neuron marker) and GFAP (astrocyte marker), and resveratrol treatment upregulated the decreased level of pCREB protein in the neurons and astrocytes of TN rats (Figures 3(f) and 3(g)).

**Figure 4:**
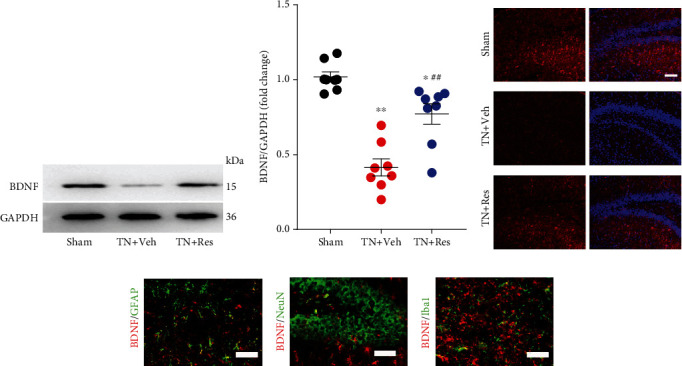
Expression and cellular distributions of BDNF protein in the DG region of the rat hippocampus. (a) Western blot showing the expression of BDNF protein in different experimental rats. (b) Quantitative analysis showing the ratio of BDNF/GAPDH in the hippocampal DG region from different experimental rats. (c) Immunostaining showing the coexpression of BDNF with DAPI in the hippocampal DG region from different experimental rats. (d) Immunostaining showing the coexpression of BDNF with GFAP in the hippocampal DG region from TN + Res rats. (e) Immunostaining showing the coexpression of BDNF with Neun in the hippocampal DG region from TN + Res rats. (f) Immunostaining showing the coexpression of BDNF with Iba1 in the hippocampal DG region from TN + Res rats. In (b), each column represents the mean ± SEM (*n* = 8 rats per group). Data analysis was performed using one-way ANOVA. ^∗^*P* < 0.05, ^∗∗^*P* < 0.01 vs. sham. ^##^*P* < 0.01 vs. TN + Veh.

BDNF is one target of CREB downstream and plays a critical role in neuropsychiatric disorders. To further investigate whether resveratrol treatment protects neurons against TN insults, a similar pattern occurred in the pCREB expression; compared to the sham group, the expression level of BDNF was decreased in the hippocampus of TN + Veh group rats, while resveratrol treatment upregulated the TN-decreased BDNF level of the hippocampus (Figures 4(a) and 4(b)). The IF results also revealed that resveratrol treatment increased the BDNF expression in the hippocampus, which was downregulated in TN + Veh rats (Figure 4(c)). Double IF revealed that the BDNF protein was highly expressed in astrocytes and partly expressed in neurons and microglial cells (Figures 4(d)–4(f)).

### 3.6. Inhibiting CREB Suppressed the Resveratrol-Induced Amelioration of Spatial Learning and Memory Impairment

**Figure 5:**
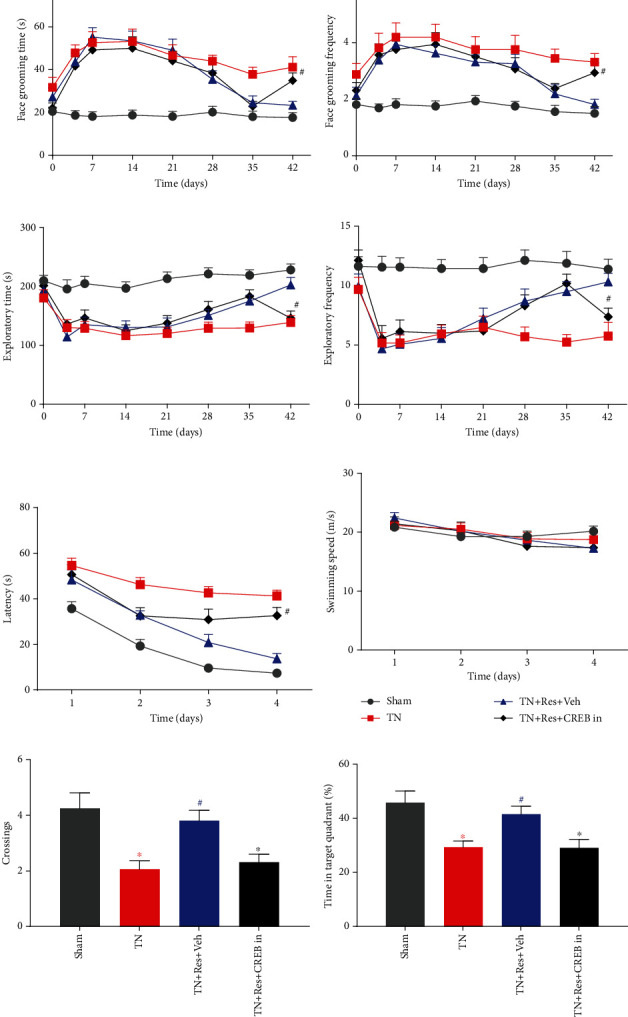
Behavioral alterations in different experimental rats after CREB inhibitor administration. (a, b) Duration (a) and frequency (b) of face-grooming behaviors at different time points. (c, d) Duration (c) and frequency (d) of exploratory activities at different time points. (e) The average escape latency in the first four days of the Morris water maze (acquisition trials). (f) Swimming speeds in the first four days of the Morris water maze (acquisition trials). (g) The number of platform site crossings in different groups during probe trials. (h) The times in the target quadrant in different groups during probe trials. Data are shown as the mean ± SEM (*n* = 16 per group). Repeated measures 2-way ANOVA with Bonferroni's post-hoc test was used to analyze behavioral alterations, latencies to reach the platform, and swimming speeds in the MWM. Data on the percentage of time spent in the platform quadrant and platform crossings were analyzed with one-way ANOVA. ^∗^*P* < 0.05 vs. sham. ^#^*P* < 0.05 vs. TN.

To investigate whether CREB activation is related to the resveratrol treatment effect on TN-induced spatial learning and memory impairment, we injected the CREB inhibitor 666–15 (10 mg/kg) into TN rats with resveratrol from POD 35-42. Compared to the resveratrol-treated TN rats (TN + Res + Veh group), CREB inhibitor injection suppressed the therapeutic effect of resveratrol on TN-induced face-grooming and exploratory activities, which were significantly reduced by resveratrol (Figures 5(a)–5(d)). To investigate whether a CREB inhibitor suppresses the resveratrol-treated improvement effect on the cognitive functions of TN rats, we used the MWM test to evaluate their spatial learning and memory abilities. As shown in Figure 5(e), during 4-day acquisition trials (POD 43-46), rats in the TN group took significantly more time to find the escape platform in the following 3 days (POD 44-46) of acquisition trials than the sham surgery group. On the other hand, rats in the TN + Res + Veh group took significantly less time to arrive at the escape platform in the trials on day 4 than rats in the TN group. After CREB inhibitor administration, the inhibition of CREB significantly suppressed the resveratrol treatment effect and increased the time required to find the escape platform more than in the TN + Res + Veh group (Figure 5(e)). Meanwhile, there was no significant difference in swimming speed among the 4 groups (Figure 5(f)). Furthermore, the TN rats exhibited a reduced number of platform site crossings and a decreased time in the target quadrant compared to the sham rats during the probe trials. Resveratrol reversed memory deterioration induced by TN, as shown by an increased number of platform site crossings and more time spent in the target quadrant in the rats of the TN + Res + Veh group than in the rats of the TN group (Figures 5(g) and 5(h)). After CREB inhibitor administration, the inhibition of CREB attenuated the resveratrol treatment effect, as demonstrated by fewer crossing numbers and less time in the target quadrant than rats in the TN + Res + Veh group (Figures 5(g) and 5(h)).

### 3.7. Inhibiting the CREB Pathway Suppressed Resveratrol-Rescued Abnormal Ultrastructural Changes in the Hippocampus and Infraorbital Nerve

**Figure 6:**
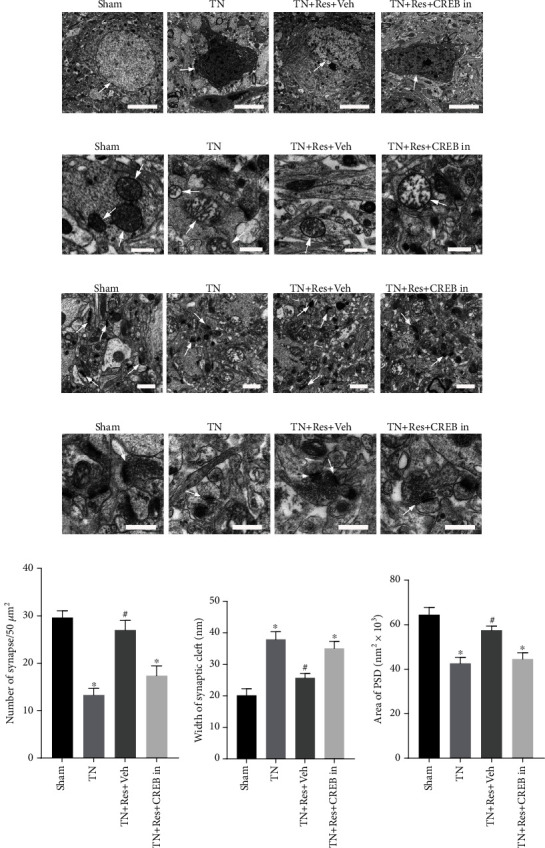
Abnormal ultrastructural changes in neurons and synapses in the DG region of the hippocampus after CREB inhibitor administration. (a) Images illustrating the differences in nuclei (white arrow) in different experimental rats (scale bars = 5 *μ*m). (b) Images illustrating the differences in mitochondria (white arrow) in different experimental rats (scale bars = 0.5 *μ*m). (c) Images illustrating the differences in synapse density in different experimental rats (magnification ×3000, scale bars = 1 *μ*m). White arrows indicate the synapses. (d) Images illustrating the differences in synapse cleft, presynaptic vesicles, and PSD in different experimental rats (magnification ×10000, scale bars = 0.5 *μ*m). White arrows show the synaptic linkages. (e) Number of synapses in the DG region of the hippocampus. (f) Width of the synaptic cleft in the hippocampal DG region. (g) Area of postsynaptic density (PSD) in the hippocampal DG region. Data are expressed as the mean ± SEM (*n* = 8 per group). Data analysis was performed using one-way ANOVA. ^∗^*P* < 0.05 vs. sham. ^#^*P* < 0.05 vs. TN.

The inhibition of CREB impairs the treatment effects of resveratrol on TN-induced spatial learning and memory deficits. Then, we investigated whether the CREB inhibitor suppressed the effects of resveratrol on the structural changes in the TN rat hippocampus. We next checked the ultrastructure changes in the hippocampus using TEM after CREB inhibitor administration. Resveratrol treatment rescued the aberrant ultrastructure of hippocampal neurons indicated by more organized nuclei, clearer organelles, and better-arranged mitochondrial cristae in rats of the TN + Res + Veh group. However, after CREB inhibitor administration, the hippocampal neurons in TN rats treated with resveratrol displayed disordered nuclear clumping of chromatin, swollen mitochondria with irregular shapes, and decreased numbers of cristae (Figures 6(a) and 6(b)). In addition, resveratrol treatment restored synaptic structures with more distinct synaptic outlines, increased synapse density, more presynaptic vesicles, narrower synaptic clefts, and thicker PSD in TN rats. Nevertheless, after CREB inhibitor administration, the TN + Res + CREB in rats exhibited a decreased synapse density with obscure synaptic outlines, fewer synapses, a wider synaptic cleft, and a thinner PSD in the hippocampus (Figures 6(c)–6(g)).

**Figure 7:**
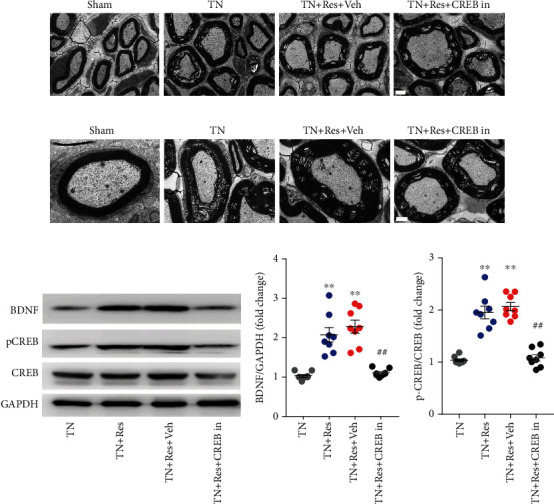
Abnormal ultrastructural changes in infraorbital nerves and protein expression of pCREB, CREB, and BDNF in the DG region of the rat hippocampus after CREB inhibitor administration. (a) Low magnification images showing the ultrastructural changes in the infraorbital nerves (ION) of rats from different experimental rats (magnification ×1500, scale bars = 2 *μ*m). (b) High magnification images illustrating the ultrastructural changes in the IONs of rats from different experimental rats (magnification ×3000, scale bars = 1 *μ*m). (c) Western blot showing the protein expression of pCREB, CREB, and BDNF in the hippocampal DG region from different experimental rats. (d, e) Quantitative analysis showing the ratio of BDNF/GAPDH and pCREB/CREB in the hippocampal DG region from different experimental rats. In (d, e), each column represents the mean ± SEM (*n* = 8 rats per group). Data analysis was performed using one-way ANOVA. ^∗∗^*P* < 0.01 vs. TN. ^##^*P* < 0.01 vs. TN + Res.

We found that TN-induced myelin sheath damage was repaired after resveratrol treatment, as shown in Figures 2(h) and 2(i). To confirm whether CREB activation is related to the resveratrol treatment effect on the repair of myelin sheath injury in TN rats, we injected the CREB inhibitor into resveratrol-treated TN rats. TN-induced severe demyelination and loss of axons in large and medium fibers of the infraorbital nerve were found to be repaired by resveratrol treatment in TN + Res + Veh group rats. As demonstrated, the ultrastructure repair of cobra venom-injured infraorbital nerve fibers was inhibited in the resveratrol treatment group of TN rats after CREB inhibitor injection (Figures 7(a) and 7(b)). The structure of myelin was disturbed, and the concentric layers expanded in the infraorbital nerve of TN + Res + CREB group rats (Figures 7(a) and 7(b)).

Furthermore, western blot results revealed that injecting a CREB inhibitor decreased the level of BDNF in the hippocampus of resveratrol-treated TN rats compared to TN + Res group and TN + Res + Veh group rats (Figures 7(c) and 7(d)). To confirm the changes in CREB signal activation after CREB inhibitor administration, we tested CREB protein expression and phosphorylation levels. Western blot results showed that CREB inhibitor administration did not change the expression level of CREB and decreased the phosphorylation level of CREB (pCREB) in the hippocampus of resveratrol-treated TN rats (Figures 7(c) and 7(e)). These results suggested that CREB inhibitor injection suppressed the improvement in spatial learning and memory impairment in resveratrol-treated TN rats by inhibiting CREB/BDNF pathway activation.

## 4. Discussion

The present study demonstrated that rats exposed to TN neuropathic pain experienced learning and memory deficits. Chronic resveratrol treatment may alleviate pain and ameliorate cognitive deficits by repairing demyelinated infraorbital nerves, restoring hippocampal neuronal and synaptic plasticity, and upregulating the CREB/BDNF pathway in the hippocampus. Nevertheless, after administration of the CREB inhibitor 666-15, neuropathic pain and learning and memory deficits were aggravated in TN + Res + CREB in rats by suppressing resveratrol-rescued abnormal ultrastructural changes and CREB/BDNF pathway activation in the TN rat hippocampus.

In the present study, we found that resveratrol treatment significantly alleviated cobra venom-induced trigeminal neuropathic pain, as demonstrated by reduced face-grooming time and frequency, increased exploratory time and frequency, and elevated ipsilateral mechanical threshold, indicating the analgesic profile of resveratrol. This finding is consistent with a previous study in which resveratrol ameliorated partial sciatic nerve ligation-induced neuropathic pain by downregulating P2X3 and suppressing the phosphorylation of ERK in the DRG [42]. Moreover, Takehana et al. also found that intravenous resveratrol alleviated trigeminal hyperalgesia by suppressing the nociceptive neuronal activity of the spinal trigeminal nucleus caudalis in rats [43].

Chronic pain, including neuropathic pain, leads to learning and memory, as well as cognitive impairments [44, 45]. The TN rats exhibited a longer latency curve, indicating impaired learning function at POD 44-46. In addition, TN rats spent less time in the target quadrant and had fewer platform crossings, demonstrating memory dysfunction at POD 47. Increasing evidence has demonstrated that resveratrol can improve various types of cognitive deficits [20, 46, 47]. The present data showed that resveratrol treatment prevented deleterious effects of pain on spatial learning and memory tested in MWW, as demonstrated by shortened latency, more crossing numbers, and longer time in the target quadrant, indicating that resveratrol prevented deficits in cognitive performance induced by pain. The data were also in accordance with previous studies showing that resveratrol improved cognitive dysfunction resulting from hippocampal neuron loss, such as in Alzheimer's disease, stress, and ischemic injury [20, 46, 47].

Neuroanatomic alterations may underlie the onset and development of cognitive impairments. Accumulating evidence indicates an overlap between the brain regions involved in cognitive changes and pain processing in patients with chronic pain [48–50]. The hippocampus plays a critical role in learning and memory. Increasing clinical studies have demonstrated hippocampal structural and functional changes in chronic pain patients [48–50]. Thus, neuronal and synaptic abnormalities in the hippocampus may contribute to pain-induced cognitive impairments. In our study, we detected reduced presynaptic vesicles and PSD thickness in synapses in the hippocampal CA1 region in rats with TN. The number of presynaptic vesicles indicates the quantity of neurotransmitters released into the cleft [51], and the thickness of the PSD represents synaptic efficacy [52]. Synaptic structural changes in the hippocampus may contribute to the memory deficits observed in our TN rats. Strikingly, 4 weeks of treatment with resveratrol restored the damaged morphologies in hippocampal neurons and synapses in rats with TN and improved their learning and memory, which also deteriorated following TN. This neuroprotective effect of resveratrol is in agreement with previous studies [53, 54], in which resveratrol delayed the progression of cognitive deficits associated with diabetes by preventing hippocampal synaptic plasticity and neuronal injury [53] and exhibited neuroprotective effects after traumatic brain injury in rats by regulating synaptic proteins and neuronal autophagy [54]. Our results, together with these findings, suggested that resveratrol improved cognitive deficits induced by chronic pain by restoring neuronal and synaptic damage.

CREB, as an important cellular transcription factor expressed in the hippocampus and prefrontal cortex [55], plays critical roles in synaptic plasticity and neuronal differentiation, which are important for memory function [56–58]. After Ser133 phosphorylation, CREB binds to cyclic AMP-response element (CRE) sequences and leads to the transcription of synaptic-related genes, such as BDNF [59], which enhances neuronal long-term potentiation (LTP) and improves cognitive function through its critical regulation of neurogenesis, dendritic length, and branching as well as synaptic plasticity [60, 61]. It was found that the CREB/BDNF pathway was downregulated in Alzheimer's disease and vascular dementia [62, 63], indicating that the expression of CREB/BDNF in the hippocampus may be associated with cognitive impairments. Consistent with our previous work, we found a decreased ratio of pCREB/CREB and BDNF expression in the hippocampus in rats with TN and cognitive deficits [9]. More importantly, we found that resveratrol increased the expression of pCREB/CREB and BDNF in TN rats and improved their cognition. Previous studies also found that resveratrol upregulated the level of pCREB and prompted the BDNF expression in the hippocampus in a chronic unpredictable mild stress (CUMS) animal model and prevented CUMS-induced cognitive deficits by regulating the CREB/BDNF expression in the hippocampus [64, 65]. These findings raise the possibility that resveratrol treatment may protect pain-induced cognitive deficits by influencing the hippocampal CREB/BDNF pathway.

Here, we revealed that pCREB was mainly expressed in the neurons and astrocytes of TN rats, and that BDNF protein was highly expressed in astrocytes and partly expressed in neurons and microglial cells. Resveratrol treatment upregulated the decreased levels of pCREB and BDNF protein in the neurons and astrocytes of TN rats and improved TN-induced cognitive deficits. Western blot analysis further demonstrated that pCREB and BDNF were markedly reduced in the hippocampus after TN, while resveratrol treatment rapidly increased the expression of pCREB and BDNF in TN rats.

To further validate whether CREB activation is related to the resveratrol treatment effect on TN-induced cognitive impairment, we injected the CREB inhibitor 666-15 into TN rats with resveratrol. We found that intraperitoneal administration of 666-15 significantly aggravated the abnormal spontaneous activities and worsened learning and memory deterioration in TN rats with resveratrol. Meanwhile, abnormal ultrastructural changes in the hippocampus were aggravated, and the CREB/BDNF pathway was decreased in the hippocampus. pCREB, BDNF, and PSD-95 decreased in the hippocampus, while CREB did not change; so, 666-15 contributed to the transmission of pain signals, suppressed the activation of the CREB/BDNF signaling pathway in the hippocampus, and weakened the treatment effect of resveratrol on cognitive disorders. There are still several limitations in our experiments; we only examined ultrastructural alterations in the hippocampus and infraorbital nerve after surgery. It is unknown whether similar alterations exist in other brain regions important for cognitive function. The CREB/BDNF pathway can be activated by various physiological and pathological stimuli. The mechanisms by which resveratrol activates the CREB/BDNF pathway in the hippocampus need to be further investigated. Additionally, we only observed morphological changes in synapses using TEM, and molecular and electrophysiological techniques will be used in the future to evaluate synapse construction and functional changes.

In summary, we demonstrated that resveratrol treatment alleviated pain behaviors and ameliorated cognitive deficits in rats with cobra venom-induced trigeminal neuralgia, possibly by restoring the ultrastructure of neurons and synapses and increasing the CREB/BDNF expression in the hippocampus. Our results suggest that resveratrol may be a potential therapeutic agent for chronic pain-induced cognitive deficits.

## Data Availability

All data will be made available by the corresponding authors upon reasonable request.

## Supplementary Materials

Supplementary Materials Supplementary Figure 1: all originally uncropped full-length gel images of western blotting of Figure 3(a) and Figure 4(a). Supplementary Figure 2: all originally uncropped full-length gel images of western blotting of Figure 7(c).
