# Supplementary material for: Resveratrol Ameliorates Trigeminal Neuralgia-Induced Cognitive Deficits by Regulating Neural Ultrastructural Remodelling and the CREB/BDNF Pathway in Rats
Source: Oxid Med Cell Longev. 2022 Nov 28;2022:4926678. doi: 10.1155/2022/4926678 (PMC9722315; doi:10.1155/2022/4926678)
Supplement: Supplementary Materials — Supplementary Figure 1: all originally uncropped full-length gel images of western blotting of Figure 3(a) and Figure 4(a). Supplementary Figure 2: all originally uncropped full-length gel images of western blotting of Figure 7(c). [file 4926678.f1.docx]

**Supplemental Files**





Supplementary Figure 1. All originally uncropped full-length gel images of western blotting of Figure 3A and Figure 4A.





Supplementary Figure 2. All originally uncropped full-length gel images of western blotting of Figure 7C.
